# Identification of Receptor Tyrosine Kinase, Discoidin Domain Receptor 1 (DDR1), as a Potential Biomarker for Serous Ovarian Cancer

**DOI:** 10.3390/ijms12020971

**Published:** 2011-01-31

**Authors:** Jinhua Quan, Tetsuro Yahata, Sosuke Adachi, Kosuke Yoshihara, Kenichi Tanaka

**Affiliations:** Division of Obstetrics and Gynecology, Niigata University, Graduate School of Medical and Dental Sciences, Niigata, 951-8520, Japan; E-Mails: zenkinka@yahoo.co.jp (J.Q.); sadachi@med.niigata-u.ac.jp (S.A.); yoshikou@med.niigata-u.ac.jp (K.Y.); tanaken@med.niigata-u.ac.jp (K.T.)

**Keywords:** ovarian cancer, tyrosine kinase, DDR1, disease biomarker, cDNA subtraction, degenerate PCR

## Abstract

Ovarian cancer, one of the most common gynecological malignancies, has an aggressive phenotype. It is necessary to develop novel and more effective treatment strategies against advanced disease. Protein tyrosine kinases (PTKs) play an important role in the signal transduction pathways involved in tumorigenesis, and represent potential targets for anticancer therapies. In this study, we performed cDNA subtraction following polymerase chain reaction (PCR) using degenerate oligonucleotide primers to identify specifically overexpressed PTKs in ovarian cancer. Three PTKs, janus kinase 1, insulin-like growth factor 1 receptor, and discoidin domain receptor 1 (DDR1), were identified and only DDR1 was overexpressed in all ovarian cancer tissues examined for the validation by quantitative real-time PCR. The DDR1 protein was expressed in 63% (42/67) of serous ovarian cancer tissue, whereas it was undetectable in normal ovarian surface epithelium. DDR1 was expressed significantly more frequently in high-grade (79%) and advanced stage (77%) tumors compared to low-grade (50%) and early stage (43%) tumors. The expression of the DDR1 protein significantly correlated with poor disease-free survival. Although its functional role and clinical utility remain to be examined in future studies, our results suggest that the expression of DDR1 may serve as both a potential biomarker and a molecular target for advanced ovarian cancer.

## 1. Introduction

Ovarian cancer, one of the most common gynecological malignancies, is an aggressive cancer associated with high morbidity and mortality, especially in the case of advanced disease. Unfortunately, ovarian cancer is rarely diagnosed in its early, most curable stages, and the tumors are already disseminated abdominally in 75% of patients at the time of diagnosis. The tumor grade, histological type, and presence of residual disease after initial surgery are also important clinicopathological factors related to patient outcome [1]. To improve the prognosis of ovarian cancer, it is vital to clarify the molecular mechanisms involved in the progression of this disease, and to develop novel and more effective treatment strategies against advanced disease.

Protein tyrosine kinases (PTKs) play an important role in the signal transduction pathways that control cell proliferation and differentiation, and are involved in tumorigenesis. Many PTKs have been shown to act as oncogenes, and analysis of PTK expression in malignant cells will lead to a better understanding of oncogenesis, which in turn may lead to novel therapies based on selective inhibition of the PTKs involved in malignant transformation [2]. Various targeted therapeutics have been explored for the management of ovarian cancer. PTKs such as Her2/neu, the epidermal growth factor receptor (EGFR), and vascular endothelial growth factor (VEGF), represent potential targets for ovarian cancer, and agents targeting these molecules are already being used in the clinic for other diseases [3–5]. The PTKs may also provide an important predictive marker for therapeutic response and patient outcome [6,7]. It is important to identify predictive PTKs to identify targeted subpopulations of patients who will respond to both the existing tyrosine kinase inhibitors (TKIs) and to new agents developed against new targets, and to obtain a better understanding of the underlying mechanisms of resistance to the existing agents so that new compounds can be developed to overcome this resistance.

The expression of PTKs can easily be determined by RT-PCR using degenerate primers which recognize common, relatively invariable cDNA sequences of members of the PTK family. PCR-based cDNA subtraction offers an efficient method for selectively amplifying differentially expressed genes. This method is particularly well-suited for the identification of target cDNAs that correspond to rare transcripts, which are typically the most difficult to obtain.

In this study, we have combined cDNA subtraction and polymerase chain reaction (PCR) using degenerate oligonucleotide primers representing conserved amino acid sequences within the tyrosine kinase domain to identify specifically overexpressed PTKs in serous ovarian cancer. The PTKs that were identified were subsequently examined for their potential association with the clinicopathological factors and patient outcome.

## 2. Materials and Methods

### 2.1. cDNA Subtraction and Degenerate PCR

Primary serous papillary ovarian cancer tissue samples and their corresponding normal ovarian tissue samples were obtained from three patients who underwent primary debulking surgery at the Niigata University Medical and Dental Hospital. Total RNA was extracted using an RNeasy Miniprep Kit (Qiagen), and poly(A)RNA was isolated using the FastTrack 2.0 Kit (Invitrogen) according to the manufacturer’s instructions. The cDNA was synthesized, and cDNA subtraction was performed for each patient using a PCR-select cDNA subtraction kit (Clontech) according to the manufacturer’s instructions. To select truly overexpressed PTKs in the cancer tissue, cDNA subtraction was performed between the tumor cDNA as a tester and 5-times the amount of normal ovarian cDNA as a driver. The subtracted cDNA fragments were amplified by suppression PCR in order to enrich the differentially expressed sequence and to reduce background. The final products of cDNA subtraction were further amplified with primers corresponding to consensus sequences for PTKs. Primers were synthesized corresponding to the amino acids HRDLAARN and DVWS(F/Y)G(I/V), which are highly conserved sequences in the catalytic domains of PTKs. The products of the degenerate PCR were subcloned and then sequenced.

### 2.2. Real-Time Quantitative Reverse Transcription Polymerase Chain Reaction Assay

Quantitative RT-PCR was performed to validate the expression of each clone identified by cDNA subtraction and degenerate PCR using Taqman probes. Tumor samples from 8 patients with serous ovarian cancer and their corresponding normal ovarian tissue were used for the analysis. Total RNA was isolated and processed with the RNeasy Miniprep Kit (Qiagen). A TaqMan two-step kit (Applied Biosystems) was utilized for all RT reactions. The High Capacity cDNA Archive kit utilizes a proprietary mixture of 10 × random primers, MultiScribe ™ Reverse Transcriptase (50 U/μL) and 10 × Reverse Transcription Buffer and 25 × dNTPs for the RT reaction. RT reactions were incubated at 25 °C for 10 min, followed by 37 °C for 120 min in a thermal cycler. After cDNA synthesis was performed during the second step, RT-PCR was then performed in an ABI PRISM 7900 in a 50 μL final volume with 1 μL of the cDNA template, 10 μM of primers and 5 μM of TaqMan Probe, and enzymes from the 2 × TaqMan Universal PCR Master Mix (ABI) according to the manufacturer’s protocol. The thermal cycling conditions were: 2 min at 50 °C, 10 min at 95 °C, followed by 40 cycles of 15 s at 95 °C and 1 min at 60 °C. The primer sequences were designed by Operon Biotechnology (Tokyo, Japan), and were as follows; IGF1-R: forward primer: 5′-ACTTCTGCGCCAACATCCTCA-3′; reverse primer: 5′-CCCTTTAGTCCCCGTCACTTCC-3′, JAK1: forward primer: 5′-AGAGGCATATAAAATTTAGATTGC-3′; reverse primer: 5′-TGTCCTTGTTGAGAGTGAACA-3′, DDR1: forward primer: 5′-ATGGAGCAACCACAGCTTCTC-3′; reverse primer: 5′-CTCAGCCGGTCAAACTCAAACT-3′, and GAPDH: forward primer: 5′-GGCTCCCACCTTTCTCATCC-3′; reverse primer: 5′-GATGTGGGGAGTACGCTGC-3′. The TaqMan-probes were obtained from Applied Biosystems.

### 2.3. Immunohistochemistry

Archival tissue from patients with ovarian cancer removed at debulking surgery and normal ovaries removed during surgery for benign conditions were used for immunohistochemical analysis. H&E-stained sections of each sample were reviewed by a pathologist, and areas corresponding to tumor tissue were marked. Immunohistochemistry (IHC) was performed on 67 serous ovarian cancer tissue specimens and 5 normal ovarian tissue specimens that were paraffin-embedded and cut into 4-μm-thick sections and mounted on positive charge-coated slides. Tissue sections were dried overnight in a 45 °C oven. For antigen visualization, the EnVision/HRP system and DAB+ (Dako) were used. The immunohistochemical procedure was optimized by testing different antigen retrieval methods using negative and positive controls. An anti-DDR1 antibody (Santacruz) was added at a 1:100 dilution to each section and incubated for 60 min at room temperature. The IHC results were scored based on the staining intensity as negative and positive. Immunoreactivity was scored as follows: the numbers of DDR1 positive cells were counted out of 100 cells in three different high power fields and judged as “positive” when >50% of cells were positively stained according to previous reports [8]. Cases were classified into two groups: group 1 (negative) included the cases with negative staining and less than 50% of staining, group 2 (positive) included cases with more than 50% of staining.

### 2.4. Association between the Expression of DDR1 Protein in Ovarian Cancer and the Clinical Disease Stage, Tumor Grade, and Patient Outcome

The clinical data for this immunohistochemical staining study were collected from 67 consecutive unselected patients with primary epithelial ovarian cancer who had undergone surgery at the Niigata University Dental and Medical Hospital between 2000 and 2004. Only patients with a histological diagnosis of serous papillary ovarian cancer were included in this study, and those with borderline tumors were excluded. All tumors were graded and staged according to the FIGO (International Federation of Gynecology and Obstetrics) classification. All patients provided consent according to the institutional review board of Niigata University Dental and Medical Hospital, Niigata, Japan.

## 3. Results

After screening ovarian cancer tissue samples by the PCR-based cDNA subtraction and degenerate PCR for selection of the clones of differentially overexpressed PTKs, we randomly selected 202 clones. By sequencing analyses, 140 clones were revealed to be identical to janus kinase 1 (JAK1), 30 to the insulin-like growth factor 1 receptor (IGF1-R), and eight to the discoidin domain receptor 1 (DDR1). The other 24 clones were unidentified genes.

**Figure 1.**
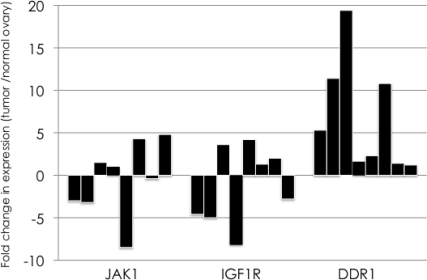
Quantitative real-time PCR for the validation of overexpression of three identified genes in ovarian cancer. Quantitative real-time PCR was performed to validate the overexpression of three identified PTKs, JAK1, IGF1R, and DDR1, in ovarian cancer. Fold change in the expression between ovarian cancer tissue and their corresponding normal ovarian tissue samples are shown with black bars. The DDR1 gene was overexpressed in all eight individuals, whereas the expression levels of JAK1 and IGF1-R were not increased in half of the ovarian cancer tissue samples.

We performed quantitative real-time PCR to validate whether the three PTKs were constitutively overexpressed in ovarian cancer using eight serous ovarian cancer tissue and their corresponding normal ovarian tissue samples. The DDR1 gene was overexpressed in all eight individuals, whereas the expression levels of JAK1 and IGF1-R were not increased in half of the ovarian cancer tissue samples (Figure 1). The DDR1 expression level in the ovarian cancer tissue samples was increased 6.7 fold on average compared with normal ovarian tissue.

**Figure 2.**
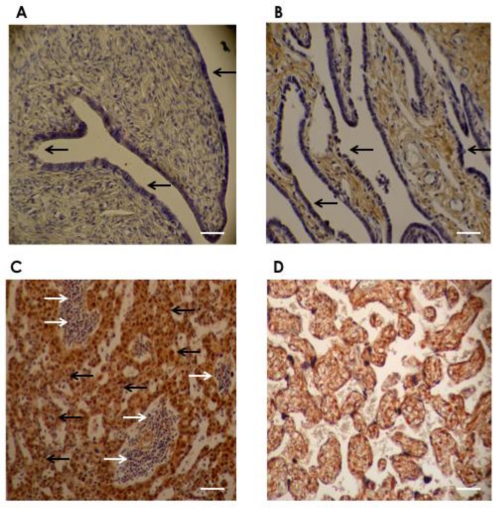
Representative immunohistochemistry staining of DDR1. Immunohistochemical analysis with the DDR1 antibody revealed negative DDR1 protein expression in (**A**) normal ovary and (**B**) serous cystadenoma, whereas positive expression was observed in (**C**) serous ovarian cancer cells. (**A**) no DDR1 staining in surface epithelium of normal ovary (black arrow); (**B**) no DDR1 staining in epithelial lining cells in serous cystadenoma (black arrow); (**C**) representative staining pattern of serous ovarian cancer tissue shows positive DDR1 staining in most serous adenocarcinoma cells (black arrow) with negative DDR1 staining in stromal cells (white arrow); (**D**) in placenta (positive control); all trophoblastic cells show positive DDR1 staining. Original magnification: ×40, scale bar: 50 μm (white bar).

To characterize the expression of DDR1, we carried out immunohistochemical analysis of 67 primary serous ovarian cancers from patients who had not received any prior treatment, obtained from primary debulking surgery. As demonstrated in Figure 2, no DDR1 staining was present in epithelial cells in normal ovary and serous adenoma, but the expression was increased in the ovarian cancer cells.

The DDR1 protein was highly expressed in 69% (46/67) of serous ovarian cancer tissue samples. Moreover, DDR1 protein expression was correlated with the pathologic grade of the tumor and the clinical disease stage at the time of surgery. As shown in Table 1, DDR1 was expressed significantly more frequently in FIGO Grade 1 (50%) compared to combined G2 and G3 (79%) tumors (*p* = 0.015).

We next compared the clinical disease stage for patients with different levels of DDR1 expression. Given the limited number of samples, the stage I and II samples were combined (early stage), as were the stage III and IV samples (advanced stage). The expression of DDR1 in early stage samples was 50%, while it was 82% in advanced stage tumors. There were significant differences in DDR1 expression found between advanced stage tumors compared with tumors found in the early stage (*p* = 0.006) (Table 2).

**Figure 3.**
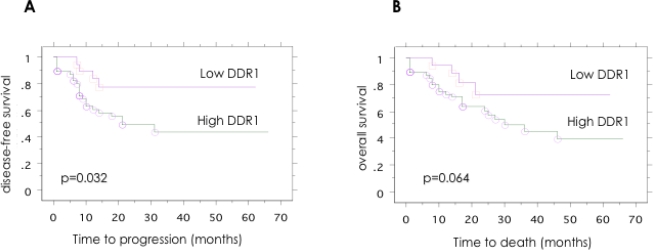
Patient outcome according to the DDR1 expression in patients with serous ovarian cancer. Kaplan-Meier analysis of (**A**) disease-free survival and (**B**) overall survival, according to DDR1 expression levels. Significant trend for shorter disease-free survival was observed in the DDR1 positive group (*p* = 0.043).

DDR1 expression was then examined for an association with disease-free survival and overall survival using Kaplan-Meier survival analysis with the log-rank statistic to determine significance. Kaplan-Meier survival curves generated for tumor DDR1, high *versus* low expression, are given in Figure 3. High tumor DDR1 expression was significantly associated with a poor outcome for disease-free survival (*p* = 0.032). With regard to overall survival, high tumor DDR1 expression showed a tendency toward a poorer outcome, but this trend was not statistically significant (*p* = 0.064).

## 4. Discussion

In the present study, we identified DDR1 as a differentially expressed PTK gene in primary epithelial serous ovarian cancer using a combination of cDNA subtraction and degenerate PCR-based cloning. DDR1 was more highly expressed in ovarian cancer samples compared with normal ovarian tissues. We were able to show that DDR1 expression is associated with the tumor grade and clinical disease stage, and is inversely correlated with the survival outcome of patients.

Receptor tyrosine kinases control a wide array of cellular responses, including the regulation of cell growth, differentiation, migration, metabolism, and survival. DDR1 was independently isolated as a novel receptor tyrosine kinase by several laboratories from human, mouse, and rat tissue in the 1990s [9–11]. DDR1 is characterized by a structural domain of 160 amino acids in its extracellular part that exhibits strong sequence similarity to the *Dictyostelium discoideum* protein discoidin 1, coagulation factors V and VIII, and to a *Xenopus laevis* recognition protein, A5. DDR1 is activated by collagen type I, II, III, V, and XI. Activation of DDR1 by collagen results in its sustained intracellular phosphorylation. DDR1 is widely expressed in epithelial cells of both fetal and adult organs. Although the physiological functions of DDR1 are not fully understood, DDR1 signaling is essential for cerebellar granule differentiation [12], arterial wound repair [13], and mammary gland development [14]. It is clear that DDR1 is involved in cell interactions with the extracellular matrix, and that it controls adhesion and cell motility [15,16].

DDR1 was found to be overexpressed in breast, brain, colon, and lung cancers, thus suggesting that this receptor may play a role in the tumorigenesis of epithelial cancers [17–20]. In breast cancer, DDR1 was overexpressed in both primary breast tumor samples and metastasis-containing lymph nodes [21]. DDR1 protein levels were elevated in 100% of patients with primary and metastatic brain tumors [18], in 61% of patients with non-small cell lung cancer, and in 64% of patients with invasive lung adenocarcinoma [22]. Thus, DDR1 expression appears to be elevated in a variety of human cancers. Consistent with studies of DDR1 in these solid tumors, elevated levels of DDR1 were seen in serous ovarian cancer in this study. Our results were also consistent with a study by Heinzelmann-Schwarz *et al.* that reported that DDR1 proteins are highly overexpressed in all histological subtypes of epithelial ovarian cancer compared with the normal ovarian surface epithelium [23].

The overexpression of DDR1 in these different human cancers suggests that it may have a function in tumor progression. It has been reported that DDR1 is overexpressed in high-grade brain, esophageal, and breast cancers, and high expression of DDR1 was associated with a significantly poorer survival in several cohorts of patients with brain, breast, and lung cancers [18,22,24–26]. In the present study, we showed that DDR1 expression was associated with high-grade and advanced stage tumors, as well as with poor survival in patients with ovarian cancer. These results were not consistent with those from previous study by Heinzelmann-Schwarz *et al.* [23]. They reported that expression of membranous DDR1 did not correlate with survival of patients. Recently Mihai C. *et al.* proposed the model of the DDR1 activation mechanism by analyzing the cellular distribution of DDR1 [27]. They showed the aggregation and cellular internalization of the receptor following collagen stimulation. In this study, we evaluated both membranous and cytoplasmic DDR1 expression and did not analyze the intensity of the staining. Although the activation process of DDR1 still remains largely unknown, evaluation of the cellular distribution or intensity of DDR1 expression might be required to analyze the precise biological activity of DDR1. Several recent studies have examined the molecular mechanisms underlying the role of DDR1 in tumor progression, invasion, and metastasis. One study showed that DDR1 expression may be regulated during the cell cycle, because overexpression of p53 in osteosarcoma cells induces DDR1 expression [28]. Ongusaha *et al.* have reported that DDR1 is a direct p53 transcriptional target, and that inhibition of DDR1 function resulted in increased apoptosis through a caspase-dependent pathway [29]. Other published data imply that the Wnt-5a pathway may overlap with DDR1 signaling [26]. Shintani *et al.* have shown that DDR1 promotes the epithelial to mesenchymal transition in response to collagen I stimulation in human pancreatic cancer cells [30]. Although experimental evidence argues against a classification of DDR1 as a transforming oncogene, subsequent steps after the initial cellular transformation, such as invasion and metastasis, might be mediated by DDR1.

One of the major characteristics of an ideal biomarker or target for molecular therapeutics is that it is absent in benign tissue and present in the targeted malignancies. Our results showed no protein expression of DDR1 in normal ovarian epithelial cells. It has been reported that DDR1 expression is highest in the brain, lungs, placenta and kidneys, and is present at low levels in various other adult tissues, such as melanocytes, the heart, liver, skeletal muscle, pancreas, and ovaries [31]. Elevated levels of DDR1 protein expression appear to be highly predictive of the presence of ovarian cancer. Although it would be nonspecific for ovarian cancer (because it could also indicate various other malignancies), the levels of DDR1 expression in body fluid or serum may have clinical prognostic utility as a biomarker for cancer.

Various targeted therapeutics have been explored for the management of ovarian cancer. In addition, numerous studies have examined the use of TKIs, including monoclonal antibodies against Her2/neu [4], other EGFRs [3], and VEGF [5], and small molecule tyrosine kinase inhibitors targeting various other receptors, including the EGFR and VEGFR [32,33]. A recent study showed that dasatinib, a multi-targeted TKI, inhibits DDR1, in addition to inhibiting BCR-ABL [34]. Moreover, DDR1 has also been identified as a potential target of other BCR-ABL inhibitors, including imatinib [35]. These inhibitors of DDR1 may prove to be therapeutically beneficial for the treatment of advanced ovarian cancer.

## 5. Conclusion

In summary, we have identified DDR1 as a differentially overexpressed PTK in ovarian cancer tissue using a combination of cDNA subtraction and degenerate PCR-based cloning. The association between DDR1 expression, tumor grade, clinical disease stage, and patient outcome suggests an *in vivo* role for this signal transduction pathway in ovarian cancer. The mechanisms by which DDR1 affects signaling cascades involved in tumor progression, invasion, and metastasis have not yet been fully characterized. Further investigation of DDR1 as a clinical biomarker and as a therapeutic target is warranted, especially for ovarian cancer.

## Figures and Tables

**Table 1.** DDR1 expression in patients with ovarian cancer according to tumor grading.

	**DDR1 positive**	**DDR1 negative**		
G1	12	12	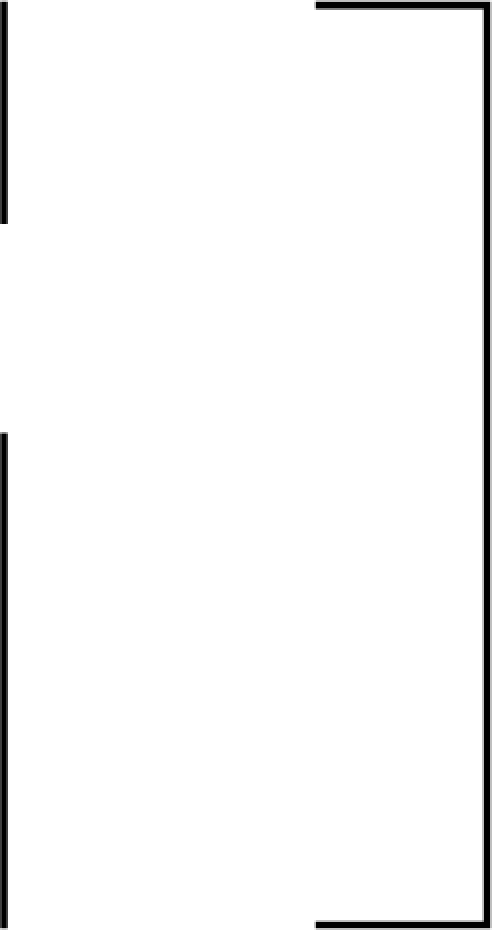	*P* = 0.015
G2	21	5		
G3	13	4		

**Table 2.** DDR1 expression in patients with ovarian cancer according to clinical staging.

**Stage**	**Cases No.**	**DDR1 positive**	**Percent of DDR1 positive**	
I	22	11	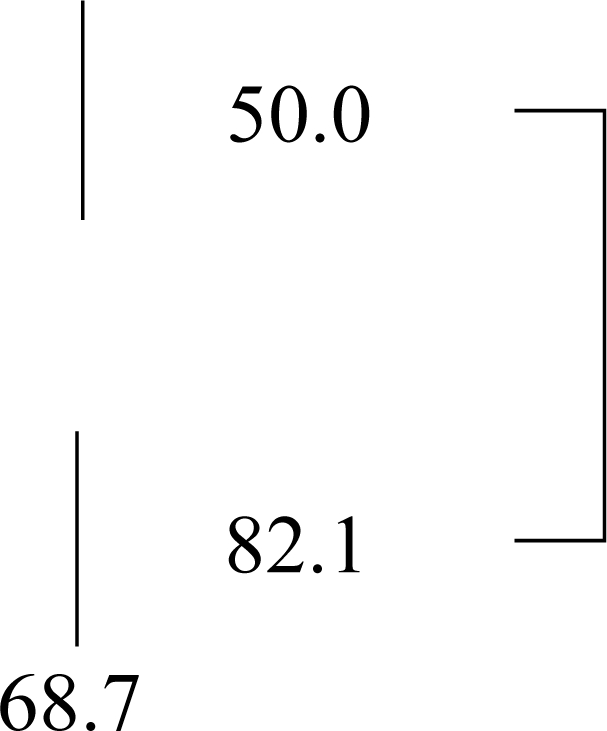	*p* = 0.006
II	6	3		
III	35	29		
IV	4	3		
total	67	46		
